# Chronic Gastritis

**Published:** 1912-08

**Authors:** W. T. Marrs

**Affiliations:** Peoria, Ill.


For Texas Medical Journal.

### Chronic Gastritis.

BY W. T. MARRS, M. D., PEORIA, ILL.

The causes of chronic gastritis, or "chronic gastric catarrh," are many and diversified. Repeated acute attacks are conducive to the disease in chronic form. It is favored by any changes in the circulation by which venous congestion of the stomach is induced, as in derangement of function in the heart, liver and spleen. Chronic gastritis is a concomitant of tuberculosis and many other depraved systemic conditions, especially anemia, chlorosis, gout, renal disease, pregnancy and the various exanthemata.

Rapid eating with faulty chewing and assimilation put a heavy tax upon the gastric mucosa, often resulting in fermentation of the stomach contents. When a person is warm, fatigued or mentally depressed and food is taken, perhaps washed down with ice water, the effects are highly damaging to the peptic and hydrochloric cells. Improper, not to say vicious, methods of eating and drinking account for much of the dyspepsia which we see so plentifully on all sides of us. Some one has said, "Indigestion is the remorse of a guilty stomach."

Another source of irritation to many occupying the more humble planes of life is the putrefaction in the mouth from carious teeth and diseased gums. Unhealthy teeth and gums cause a constant oozing of toxic material into the stomach. Alcohol and tobacco are also strongly conducive to the large contingent of derelict stomachs.

The clinical signs of chronic gastritis are, as a rule, sufficiently clear to enable one to arrive at a reasonably correct diagnosis without scientific aids. However, the examination of the stomach contents following a test-meal affords excellent means for accuracy and fine points in the diagnosis. As this procedure is so fully set forth in any standard work on diseases of the digestive tract, I feel that my entering into same in this brief article would be another case of "carrying coals to Newcastle."

Several other pathological conditions should be carefully considered and excluded in making a diagnosis. Especially should we exclude cancer, ulcer, dilatation, neuroses and the various acute derangements of the stomach. Any of these may produce symptoms similar to chronic gastritis; on the other hand, a stubborn gastritis may to a degree simulate any of the above named disorders.

The tongue is usually coated but lacks the thick fur which accompanies carcinoma. In anemic conditions the tongue presents a transparent, bluish appearance. Gaseous eructations are frequent. Pyrosis and cardialgia are common, and these symptoms must be distinguished from gastralgia; the latter is a diffuse pain in the stomach. In case of sour stomach and vomiting it is well to ascertain whether this be due to hypersecretion of hydrochloric acid or to a fermentation from other acids, as fatty, lactic, acetic, etc. The appetite varies, usually fickle and capricious but sometimes voracious. Food may cause a vague, choking sensation in the epigastrium with pains of a neuralgic character. Another annoying symptom comes from the effort of the oppressed stomach to drive the ingesta into the intestines.

A later manifestation relates to a weakness of the gastric muscular coat, or atony of the stomach, which in turn causes a lengthened stay of food in the stomach. It is in this condition that we are especially likely to find distension of the stomach, with troublesome gaseous eructations, sour stomach, etc. Constipation and hepatic insufficiency are the rule. The urine is scanty, deposits urates abundantly, and it is at times alkaline from basic salts. Flatulence with rumbling in the intestines are common. Hard masses of fecal matter—scybila—are often found in the rectum, and this may lead to a paresis of the muscular coat of the bowel as well as produce piles, fissures, etc. The victims of chronic gastritis are by no means always lank, lean and poorly nourished; often such sufferers are rotund and apparently well nourished.

The treatment should aim at the correction of the depressed state of the stomach and the regulation of the patient's habits, especially with reference to eating and drinking, and the auxiliary aids to digestion should have appropriate attention. The dietary should be gone over carefully with the patient and such articles as he may manifest an idiosyncrasy for should be judiciously interdicted or restricted. As a rule, it is not so much what the patient eats that hurts him as the quantity and the improper manner of partaking thereof.

An occasional lavage of the stomach with a normal salt solution may give good results. The virtue of the lavage consists in the removal from the stomach of remnants of food which have remained there unduly long, as well as loosening the mucus which adheres to the stomach walls. Furthermore, lavage increases peristalsis and strengthens muscular activity and influences glandular action favorably.

Peptenzyme has given me the promptest results of remedies employed for direct relief. It is more than a pepsin and pancreatin preparation, and in addition to these ferments it contains the nucleo-enzymes of the hepatic, salivary and intestinal glands as well as of the pancreas and spleen. It therefore acts not only as a digestant but also as a corrective of the secretory function. Two tablets should be administered whenever food is taken. Thus employed in conjunction with other indicated remedies and measures it has always given me gratifying results.

Stomachics like nux vomica, gentian and quassia are at times of supplementary aid. Laxatives and hepatic stimulants that are occasionally of value are cascara, calomel and salines. A colonic flushing may be of service when hardened fecal matter accumulates in the sigmoid flexure and descending colon.

